# Env7p Associates with the Golgin Protein Imh1 at the *trans*-Golgi Network in *Candida albicans*

**DOI:** 10.1128/mSphere.00080-16

**Published:** 2016-08-03

**Authors:** Kongara Hanumantha Rao, Swagata Ghosh, Asis Datta

**Affiliations:** a National Institute of Plant Genome Research, New Delhi, India; b Department of Molecular Biology and Biotechnology, University of Kalyani, West Bengal, India; Yonsei University

**Keywords:** *Candida albicans*, Env7, Imh1, *trans*-Golgi network, palmitoylation, virulence

## Abstract

Vesicular dynamics is one of the very important aspects of cellular physiology, an imbalance of which leads to the disorders or diseases in higher eukaryotes. We report the functional characterization of a palmitoylated protein kinase from *Candida albicans* whose homologue in *Saccharomyces cerevisiae* has been reported to be involved in negative regulation of membrane fusion and was named Env7. However, the downstream target of this protein remains to be identified. Env7 in *C. albicans* (*Ca*Env7) could be isolated from the membrane fraction and localized to vesicular structures associated with the Golgi apparatus. Our work reports Env7 in *C. albicans* as a new player involved in maintaining the functional dynamics at the *trans*-Golgi network (TGN) by interacting with two other TGN-resident proteins, namely, Imh1p and Arl1p. Direct interaction could be detected between Env7p and the golgin protein Imh1p. Env7 is itself phosphorylated (Env7p) and phosphorylates Imh1 *in vivo*. An interaction between Env7 and Imh1 is required for the targeted localization of Imh1. *Ca*Env7 has a putative palmitoylation site toward both N and C termini. An N-terminal palmitoylation-defective strain retains its ability to phosphorylate Imh1 *in vitro*. An *ENV7* homozygous mutant showed compromised filamentation in solid media and attenuated virulence, whereas an overexpressed strain affected cell wall integrity. Thus, Env7 plays a subtle but important role at the level of multitier regulation that exists at the TGN.

**IMPORTANCE** A multitier regulation exists at the *trans*-Golgi network in all higher organisms. We report a palmitoylated protein kinase, Env7, that functions at the TGN interface by interacting with two more TGN-resident proteins, namely, Imh1 and Arl1. Palmitoylation seems to be important for the specific localization. This study focuses on the involvement of a ubiquitous protein kinase, whose substrates had not yet been reported from any organism, as an upstream signaling component that modulates the activity of the Imh1-Arl1 complex crucial for maintaining membrane asymmetry. Virulence is significantly diminished in an Env7 mutant. The functioning of this protein in *C. albicans* seems to be quite different from its nearest homologue in *S. cerervisiae*, which reflects the evolutionary divergence between these two organisms.

## Abstract (precis)

A multitier regulation exists at the *trans*-Golgi network in all higher organisms. We report a palmitoylated protein kinase, Env7, that functions at the TGN interface by interacting with two more TGN-resident proteins, namely, Imh1 and Arl1. Palmitoylation seems to be important for the specific localization. This study focuses on the involvement of a ubiquitous protein kinase, whose substrates had not yet been reported from any organism, as an upstream signaling component that modulates the activity of the Imh1-Arl1 complex crucial for maintaining membrane asymmetry. Virulence is significantly diminished in an Env7 mutant. The functioning of this protein in *C. albicans* seems to be quite different from its nearest homologue in *S. cerervisiae*, which reflects the evolutionary divergence between these two organisms.

## INTRODUCTION

Cellular membranes that form the barriers and partition all eukaryotic cells into distinct compartments undergo remodeling through fusion and fission events (1). Such events are influenced by intrinsic and extrinsic forces to generate curved structures that get associated with diverse cellular architectures. The Golgi apparatus is one such organelle in which coat complexes along with coiled-coil proteins that trap vesicles interact with each other and generate membrane curvature. The *trans*-Golgi network (TGN) refers to a membranous compartment located on the *trans* side of this stacked cisterna, which sorts Golgi apparatus products according to their final destinations through vesicular transport. This remarkably sophisticated process of vesicular transport is widely conserved from humans to yeasts (2) and relies on transport vesicles that are formed from one membrane compartments and fuse with another to release associated cargo molecules (3). Critical regulation is exerted on several aspects of this entire phenomenon, one of the most important being in the level of membrane curvature.

We report a widely expressed serine-threonine kinase as a new member of the TGN protein cascade involved in fusion-fission dynamics. This has been known by different names as protein kinase expressed in day 12 fetal liver (coded for by *PKL12*), kinase related to *Saccharomyces cerevisiae* and *Arabidopsis thaliana* (coded for by *KRCT*), embryo-derived protein kinase (coded for by *EDPK*), and myristoylated and palmitoylated serine-threonine kinase (coded for by *MPSK1*) (4) and has been described recently in *Saccharomyces cerevisiae* as the product of *ENV7* (5). In *S. cerevisiae*, Env7 has been described as a palmitoylated protein kinase that negatively regulates membrane fusion. We show the protein to be involved in maintaining a subtle dynamic at the *trans*-Golgi network in the human pathogen *Candida albicans*. This finding also emphasizes a more ubiquitous occurrence of this protein across different organisms.

Although this protein has been recently reported from various organisms like *S. cerevisiae*, *A. thaliana*, and humans, its downstream target could not be identified until date. Our data suggest interactions between Env7 and a golgin protein, Imh1, and ADP ribosylation factor-like protein (Arl1). Imh1 has been reported to be an effector of Arl1 and is targeted to the Golgi apparatus via interaction of their GRIP domain with GTP-bound form of Arl1 (6–8). We show that Env7 directly interacts with Imh1 and phosphorylates it. A recent study by Tsai et al. (9) concluded their work by stating that “the challenge will now be to further elucidate the exact order and regulation of the dynamic and multiple interactions between Arl1p and its binding effectors in regulating the structure and function of the TGN.” Thus, Env7, an upstream kinase in this pathway in *C. albicans*, will add new information about the relay mechanism and help in better understanding of the complex regulatory control at the TGN. A dysregulated localization of Imh1 was observed in an *env7* mutant, indicating that Env7 by associating with Imh1 affects its Golgi apparatus association status. Furthermore, a *C. albicans* env7 mutant showed reduced filamentation and attenuated virulence in murine model. Overall, the study of this protein could be important in understanding diseases related to TGN dysfunction.

## RESULTS

### *Ca*Env7 is localized to the *trans*-Golgi.

**FIG 1:**
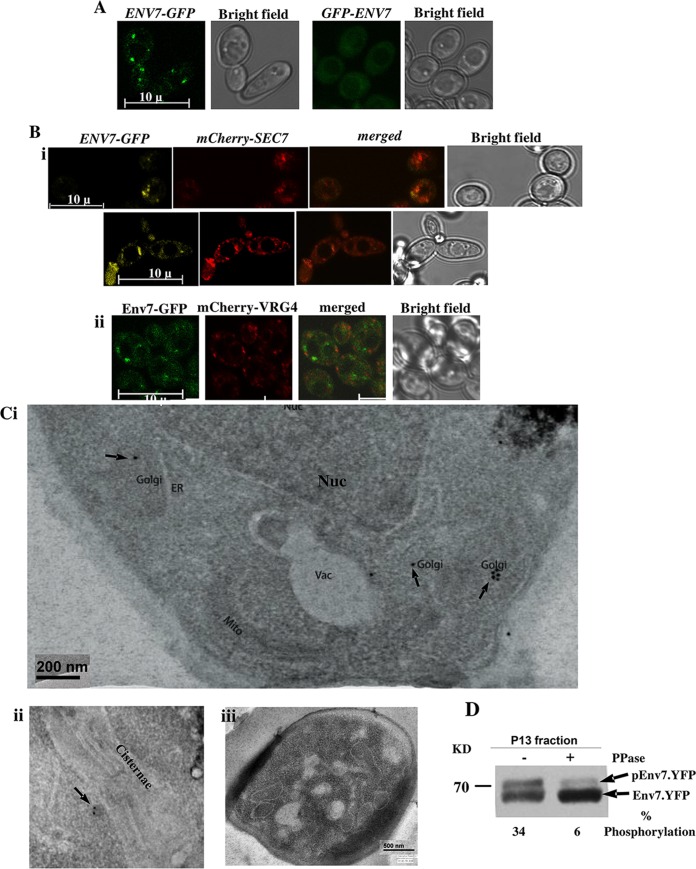
Env7 in *Candida albicans* localizes to the *trans*-Golgi network and is a phosphorylated protein. Env7 in *Candida albicans* localizes to the *trans*-Golgi network and is a phosphorylated protein. (A) A C-terminally tagged Env7 protein under the *ADH1* promoter (strain EN-pYPB-ENV7-GFP) localizes at Golgi apparatus-like punctate structures. An N-terminal tagging of the same protein under the *ADH1* promoter (strain EN-pYPB-GFP-ENV7) results in mislocalization. (Bi) The wild-type *C. albicans* strain expressing Env7 was cotransformed with mCherry-SEC7 under the *ADH1* promoter and imaged using confocal microscopy. Representative GFP-tagged Env7, mCherry-tagged Sec7, merged images, and bright-field images are shown. (Bii) The wild-type *C. albicans* strain expressing Env7 was cotransformed with mCherry-VRG4, under the *ADH1* promoter and imaged using confocal microscopy. Representative GFP-tagged Env7, mCherry-tagged Vrg4, merged images, and bright-field images are shown. Merging could not be observed between the two proteins. (Ci) An immunoelectron microscopic (IEM) image shows endogenous Myc-tagged Env7 (in the ENV7-Myc strain) immunolabeled with gold particles. Arrows indicate clusters of immune gold particles associated with the Golgi apparatus. The organelles are labeled: Nuc, nucleus; Golgi, Golgi apparatus; Mito, mitochondria; Vac, vacuole; ER, endoplasmic reticulum. (Cii) Zoomed-in image of vesicular structures budding off from cisternae. (Ciii) Negative control using an untagged strain undergoing all of the downstream processing of IEM. (D) A P13 membrane fraction shows bands, one of which shows reduced migration. The intensity of the upper band was reduced upon incubation with alkaline phosphatase.

Fluorescence of a green fluorescent protein (GFP)-tagged *Ca*Env7 (Env7-GFP) strain using an endogenous promoter remained undetected. Env7 with a C-terminally tagged GFP and expressed under an *ADH1* promoter showed localization in distinct punctate structures resembling localization of Golgi proteins in yeast (Fig. 1A). However, an N-terminally tagged *Ca*Env7 fusion protein was mislocalized to the cytoplasm (Fig. 1A), in contrast to *S. cerevisiae*, where Manandhar et al. reported that localization is independent of tag orientation (5). Coexpression of Env7-GFP with the *trans*-Golgi marker mCherry-Sec7 confirmed the punctate dots to be *trans*-Golgi (Fig. 1B). To strengthen the specificity of Env7 localization to the *trans*-Golgi and rule out the possibility that Env7 is a general component of the Golgi apparatus, we have performed colocalization studies with *cis*-Golgi marker Vrg4 and found that Env7-GFP and mCherry-Vrg4 are not merging with each other (Fig. 1Bii). Further confirmation was done by performing immunoelectron microscopy with an Env7-13×Myc endogenously tagged strain. Colloidal gold particles localized to vesicles budding off from the *trans* phase of Golgi stacks (Fig. 1C). To biochemically confirm its membrane association, the total membrane fraction (P13) was isolated as described in Materials and Methods. In a low-percentage gel, Env7 of the membrane fraction migrated as two closely spaced bands, the upper of which decreased upon protein phosphatase treatment. Thus, Env7 is a phosphorylated, membrane-associated protein (Fig. 1D).

To confirm the functionality of all tagged forms of Env7 proteins, their ability to form filaments was checked on a filamentation-inducing medium, like Spider medium (see Fig. S1 in the supplemental material).

10.1128/mSphere.00080-16.1 FIGURE S1  Functionality of all tagged forms of Env7 proteins is confirmed by ability of colonies to form filaments on Spider medium. Download FIGURE S1, TIF file, 0.7 MB. Copyright © 2016 Rao et al. 2016 Rao et al. This content is distributed under the terms of the Creative Commons Attribution 4.0 International license.

### *Ca*ENV7 is a palmitoylated protein, and its palmitoylation is required for its specific localization.

**FIG 2:**
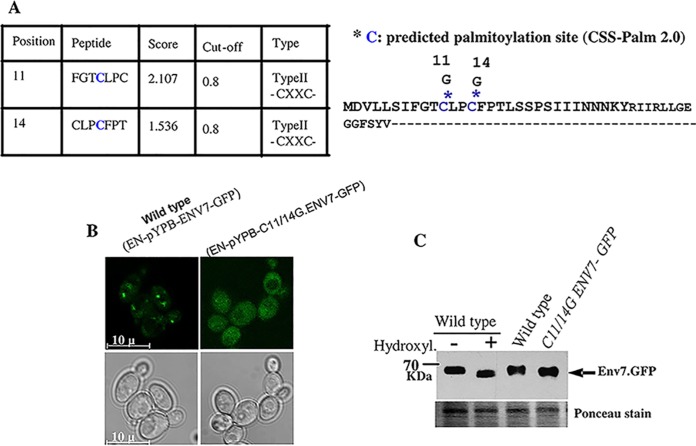
Palmitoylation affects Env7 localization. (A) Palmitoylation sites (left panel) were predicted (*in silico* prediction) by using CSS-Palm 2.0 software (36). The letters in blue represent predicted palmitoylation sites. Positions 11 and 14 are with high-threshold values. The cysteine residues (blue) modified to glycine residues by site-directed mutagenesis (SDM) to prevent palmitoylation are shown with asterisk (right panel). (B) Env7p-GFP is localized in the cytoplasm in C11/14G-ENV7 strain (EN-pYPB-C11/14G.ENV1-GFP). Modification of cysteine residues or prevention of palmitoylation resulted in localization of Env7-GFP in the cytoplasm. (C) Western blot showing change in the migration between wild-type Env7p-GFP fusion protein and C11/14G.ENV1-GFP proteins along with hydroxylamine-treated sample. Wild-type and SDM strains were grown in YPD for 6 h at 30°C, washed in sterile water, and induced in SD for 1 h. Western blot analysis was carried out using 15 µg of protein from each sample to assess protein migration. Ponceau-stained bands are shown as a loading control.

In general, Golgi apparatus- or vesicular membrane-localized proteins possess either transmembrane domains or posttranslational modifications to get docked to membranes (10). Our analysis for the detection of transmembrane domains (TMDs) by using DAS transmembrane prediction software (see Fig. S2 in the supplemental material) resulted in a profile atypical for membrane-spanning proteins. Hence we hypothesized that some posttranslational modification could be attributed to its membrane association. In a global analysis of protein palmitoylation in *S. cerevisiae* using the MUDPIT-MS method, Roth et al. detected YPL236C, the *C. albicans* Env7 homologue, as the sole palmitoylated protein in the category where cysteine residues are located near the N terminus (10). This was quite interesting since palmitoylation sequences are almost always located at the carboxy termini of proteins. Interestingly in *C. albicans*, the predicted palmitoylation sites are present toward both the N and C termini as revealed by CSS Palm2.0 software, which could be an evolutionarily favored phenomenon over its *Saccharomyces* homologue (Fig. 2A; see Fig. S3A and B in the supplemental material) by preventing palmitoylation by site-directed mutagenesis of the two N-terminal cysteine residues to glycine (Fig. 2A, right panel), we were able to show that palmitoylation of *Ca*Env7 is crucial for its specific subcellular localization as the mutant showed a diffused distribution throughout the cytoplasm and a complete absence of punctate spots (Fig. 2B). At this point, we cannot rule out the possibility that site-directed mutagenesis of the N-terminal palmitoylation residues can influence simultaneous modification at the C-terminal residue. Therefore, we hypothesize that palmitoylation affects localization of this protein.

10.1128/mSphere.00080-16.2 FIGURE S2  Transmembrane domains (TMDs) determined by using DAS transmembrane prediction software. Download FIGURE S2, TIF file, 0.1 MB. Copyright © 2016 Rao et al. 2016 Rao et al. This content is distributed under the terms of the Creative Commons Attribution 4.0 International license.

10.1128/mSphere.00080-16.3 FIGURE S3  Palmitoylation sites predicted by using CSS-Palm 2.0 software (A) or CSS-Palm GPS-CUCKOO (B) (*in silico* prediction). Download FIGURE S3, TIF file, 0.4 MB. Copyright © 2016 Rao et al. 2016 Rao et al. This content is distributed under the terms of the Creative Commons Attribution 4.0 International license.

The membrane fraction was isolated from both wild-type (WT) and C11- and 14G-Env7-GFP strains and subjected to *N*-ethylmaleimide treatment followed by hydroxylamine treatment to release thio-ester-linked palmitoyl moieties. Western blot analysis revealed a slightly faster migration of the Env7 band in both the C11- and 14G-Env7-GFP strains (without hydroxylamine treatment) and the wild-type strain treated with hydroxylamine (Fig. 2C). This further confirmed that Env7 was a palmitoylated protein. The significance of predicted C-terminally palmitoylated sites is under investigation.

In an earlier section, we pointed out that GFP-Env7 (N-terminally tagged) was distributed in the cytoplasm. This was quite a contrast from what had earlier been reported for the homologue of this protein in *S. cerevisiae* (5). This altered localization of the N-terminal fusion protein led us to two possible hypotheses. The first one is that an N-terminal GFP tag prevents the accessibility of the cysteine residues for palmitoylation by palmitoylation machinery. The second one is that even after palmitoylation, the palmitoylated residues are not available for membrane tethering due to the presence of a GFP moiety toward the N terminus. Hydroxylamine treatment of the GFP-Env7 strain showed that palmitoylation occurred in the cysteine residues, but these residues are not available for membrane docking. Therefore, we hypothesized that it is the tethering function that got disturbed in an N-terminal fusion protein.

### Characterization of *env7* mutant.

**FIG 3:**
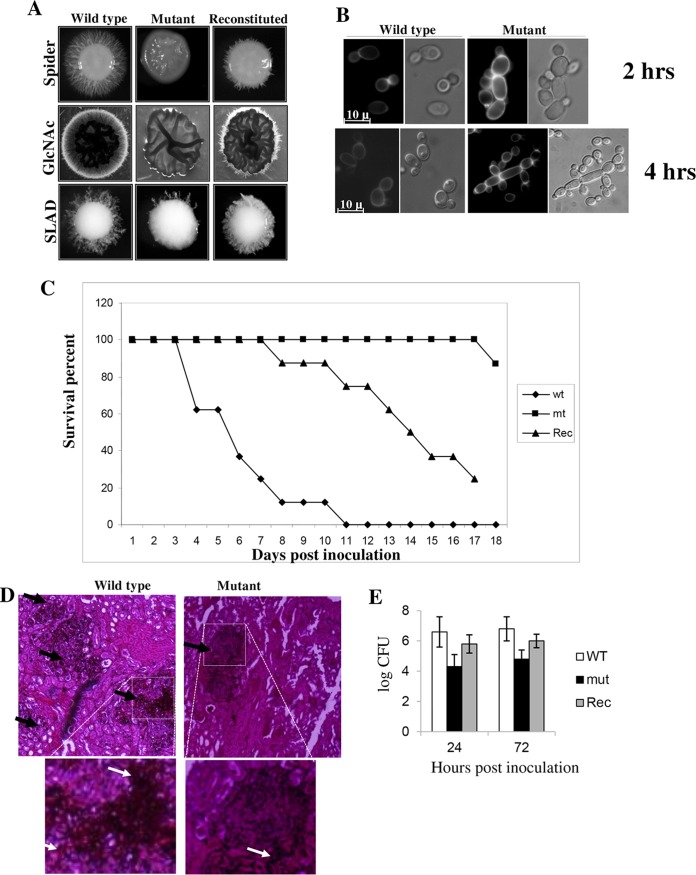
Characterization of *env7* mutant. (A) The *Caenv7* mutant showed reduced filamentation on solid filamentation-inducing media. Hypha formation on Spider, GlcNAc, and SLAD plates is shown. Strains were incubated at 37°C for 7 days in the case of Spider medium, 5 days in the case of GlcNAc, and 10 days in the case of SLAD plates. The wild-type strain (SN-HLA), *Caenv7* mutant (EN102), and complemented strain (EN103), in which one functional copy of *Caenv7* was reintroduced in the native locus, are shown. (B) Mutant cells grown in YPD liquid form chains. Shown are the morphologies of wild-type (SN-HLA) and *env7* mutant (EN102) cells grown in YPD for 2 h and 8 h at 30°C. The mutant strain showed chains of cells at the 2-h time point, and with increased time, more pseudohyphae were observed. Cell wall thickenings are shown by arrows after staining with calcofluor white. (C) Effect of *Caenv7* mutation on *C. albicans* virulence. Infection of mice with a *Caenv7* strain leads to enhanced survival. Mice were infected intravenously with 5 × 10^6^ cells of the wild-type strain (SN-HIS1), *Caenv7* mutant (EN102), and *Caenv7* reconstituted (Rec) strain (EN103). Data from one experiment using five mice are shown and are representative of three identical, separate experiments. The results were statistically significant (*P* < 0.01, *t* test). (D) Periodic acid-Schiff’s staining of kidney sections from mice infected with wild-type or the *env7* mutant strain (EN102) 3 days postinjection. Pictures were taken at ×20 magnification. The focal lesions formed by fungus are represented with black arrows, and these lesion numbers are higher in the WT (SN-HLA) than in the mutant (EN102). The lower panels show magnifications of the boxed areas in the upper panels. White arrows represent filaments within the tissue. (E) Representation of the quantitative fungal burden in the kidney measured by serial dilution and expressed as log CFU per gram of tissue (*y* axis). Subgroups of 3 to 5 mice were sacrificed at 24 or 72 h after infection. Data are represented as means of standard deviations from three separate experiments with 3 to 5 mice per strain (*P* < 0.01, *t* test).

Among the proteins that rely on palmitoylation for specific localization are many of the key players that are involved in cellular signaling and can further affect cell physiology and morphology. Keeping this in mind, we investigated the effect of disruption of *ENV7* in the dimorphism of *C. albicans* on plates containing established media like Spider GlcNAc and SLAD (defined below) (Fig. 3A). The homozygous mutants were impaired in hypha formation to some extent under all of the conditions mentioned above and were mostly smooth at the periphery. When vegetatively grown in liquid yeast extract-peptone-dextrose (YPD), homozygous mutant cells showed some interesting morphology and were slightly elongated and tended to aggregate. Initially, the cells grew as short chains, which aggregated within 2 h, and by 8 h after inoculation, most of the cells in each population had assumed a polarized, pseudohyphal mode of growth, and there were obvious constrictions at the sites of septation (Fig. 3B). These defects in solid and liquid media were reversed by reintroduction of the *CaENV7* gene.

In order to investigate whether *ENV7* is required for virulence, mice were inoculated intravenously with wild-type, mutant, and complemented *C. albicans* cells (5 × 10^6^ cells) and monitored for survival. Over the 21-day observation period, one mouse died after day 18 in the group infected with the homozygous mutant strain. In contrast, the median survival times of mice infected with the wild-type strain and the complemented strain were around 5 and 11 days, respectively (Fig. 3C). We reproduced the infection experiment two more times with groups of 5 mice per *C. albicans* genotype, essentially yielding identical results. The results unequivocally demonstrate an attenuated virulence in *Caenv7* mutant cells. Histological sections of kidney recovered from wild-type-infected mice and stained with periodic acid-Schiff’s stain showed numerous focal collections of *Candida* filaments, whereas sections from the mutant had very few areas of infection (Fig. 3D). To evaluate whether the reduced virulence of the *env7* strain was due to its reduced multiplication *in vivo*, we conducted a parallel set of experiments in which mice infected with either the WT or the mutant were sacrificed, and the level of fungal colonization of the kidney was determined. High fungal burdens were observed in WT-infected mice from 24 h to 72 h postinfection. CFU counts from the kidneys of mice infected with the *env7*strain were significantly lower than those for WT-infected mice (Fig. 3E). Overall, the reconstituted strain could not restore completely wild-type morphology or virulence and showed an intermediate response (Fig. 3A and C) because of the phenomenon of haploinsufficiency displayed by the fungus (11).

### An increased level of Env7 makes cells susceptible to cell-wall-perturbing agents.

**FIG 4:**
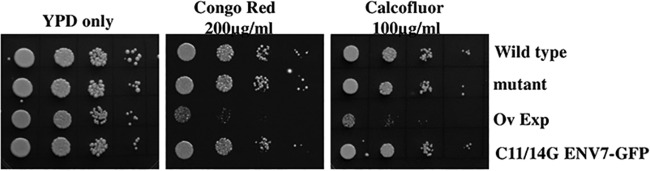
Growth analysis of mutant and *CaENV7*-overexpressing strains. Sensitivity to stress-inducing conditions. The wild-type, *env7* mutant, *ENV7-*overexpressing (IpGAL-ENV7), and EN-pYPB-C11/14G.ENV7-GFP strains were grown in YPD for 8 h at 30°C, the cell concentration was adjusted to an OD of 1, and 3 µl of the 10-fold dilution series was spotted on plates containing YPD, YPD plus Congo red, and YPD plus calcofluor white with the indicated concentrations of cell-wall-perturbing agents.

Tinkering with proteins of the Golgi network often compromises protein glycosylation, cell wall biogenesis, and cell integrity (12–14). Interestingly the *env7* mutants grew like the wild type in Congo red or calcofluor plates, whereas the overexpressed strain showed hypersensitivity to these reagents (Fig. 4). This suggests that although the deletion does not affect cell wall integrity, overexpression became deleterious for the cell.

### *Ca*Env7 interacts with Imh1-Arl1 complex.

**FIG 5:**
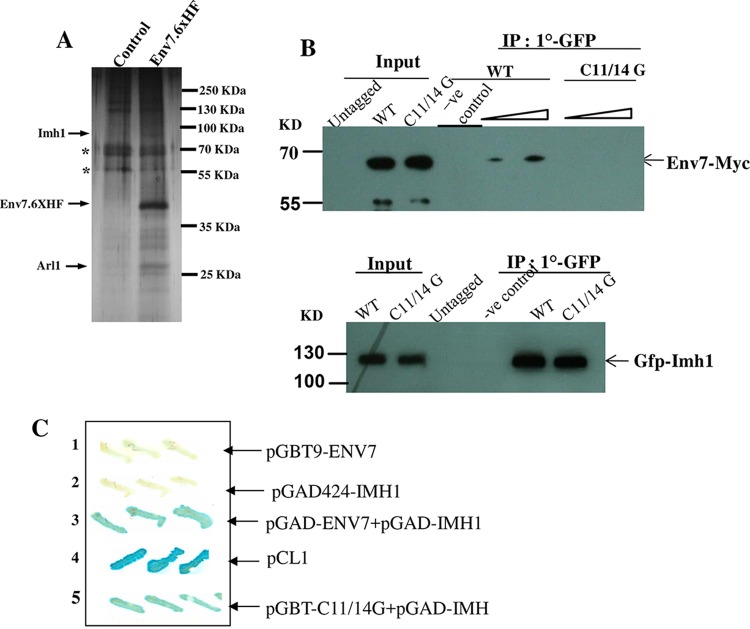
Env7 interacts with Imh1 *in vivo*. (A) *C. albicans* cells expressing Env7-6HF were subjected to TAP procedures as described in Materials and Methods using anti-Flag and Ni-NTA agarose. Solid arrowheads represent the components of Env7 complex identified through matrix-assisted laser desorption ionization–time of flight (MALDI-TOF). BWP17 (control) and ENV7-6HF (TAP-tagged ENV7) cells were grown in YPD containing 0.6 M NaCl. Nonspecific bands are represented with asterisks. The TAP purification was repeated four times. (B) Env7 interaction with Imh1 was confirmed by coimmunoprecipitation. The strains BEI71 and BEI71-C11/14G (Env7 wild type or Env7-C11/14G tagged with Myc tag expressed under native promoter and IMH1 N-terminally tagged with GFP and expressed under GAL1 promoter) were induced in YP-galactose, and crude extract was isolated as described in Materials and Methods. Five percent of the crude extract was used as input. Immunoprecipitation was carried out by using anti-GFP antibody and protein A/G Sepharose beads. Four-fifths of the immunoprecipitated sample was probed with anti-c-Myc antibody (upper panel), and remaining 1/5th was loaded on separate gel and probed with anti-GFP antibody (lower panel). As a negative control, crude lysate of the Env7-Myc-tagged strain was subjected to pulldown assay as described above. (C) Two-hybrid assay. The ENV7 and IMH1 genes were cloned in pGBT9 (GAL4 DNA binding domain) and pGAD424 (GAL4 activation domain), respectively, and transformed in yeast strains. Lane 1, only pGBT9-ENV7; lane 2, only pGAD424-IMH1; lane 3, both pGAD-ENV7 and pGAD-IMH1; lane 4, positive-control vector-pCL1; lane 5, both pGBT-C11/14G and pGAD-IMH1. The yeast two-hybrid assay revealed that both the Env7 wild type and Env7-C11/14G physically interact with Imh1.

To identify the downstream effectors of *Ca*Env7, we performed protein complex purification by using a tandem affinity purification (TAP) strategy. After a second round of purification and detection through liquid chromatography-mass spectrometry (LC-MS), the occurrence of Imh1 and Arl1 was consistently associated with Env7 (Fig. 5A). To further confirm this interaction of Env7 with Imh1, we have performed coimmunoprecipitation (co-IP) with the strain carrying Env7-13×Myc and GFP-Imh1. Using anti-GFP antibody and protein A/G Sepharose followed by Western blotting with anti-c-Myc antibody, Env7 could be detected (Fig. 5B, upper panel). To out rule nonspecific interactions, crude lysate from the Env7-13×Myc-tagged strain was subjected to pulldown under conditions similar to those described above. In parallel, to assess the immunoprecipitation efficiency, a fraction of the same immunoprecipitant was probed with anti-GFP antibody in a separate blot. An Imh1 band was detected, which proved that IP was working (Fig. 5B, lower panel). However, we could not detect the signals for Env7-Myc in the immunoprecipitants carried out from the cell lysates obtained from C11/14G Env7-Myc strain (Fig. 5B, upper panel).

To check whether Env7 and Imh1 showed a physical interaction, a two-hybrid assay was performed with both wild-type and C11/14G versions of Env7. Interestingly, a positive physical interaction was observed in both cases (Fig. 5C). Therefore, the C11 and 14G residues are not needed for the interaction between Imh1 and Env7 but contribute to the colocalization of the two interacting proteins.

### Env7 phosphorylates Imh1 and is required for the normal localization of Imh1.

**FIG 6:**
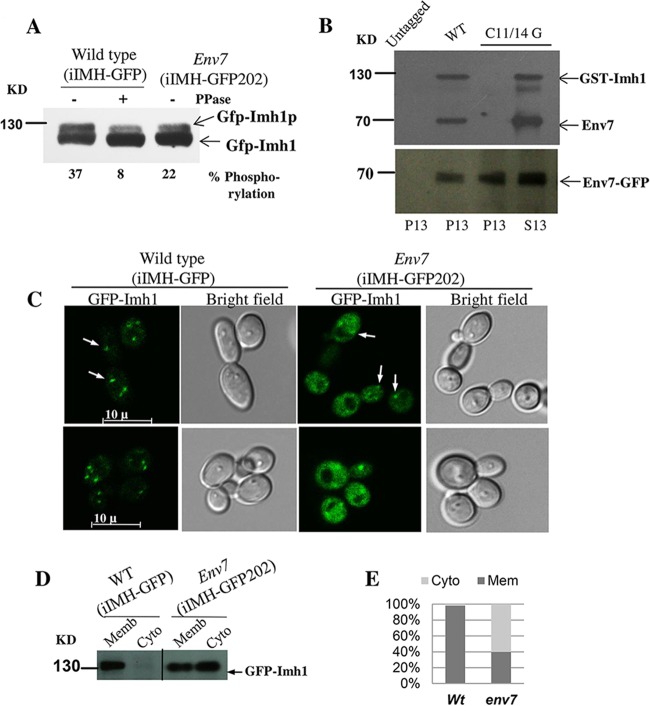
Env7 phosphorylates Imh1 *in vivo*. (A) The membrane fraction of the strains iIMH-GFP (wild type) and iIMH-GFP202 (*env7* mutant) were collected and checked for upshifted bands in a low-percentage gel (6.5%) in the absence or presence of protein phosphatase. Bands of lower-exposure blots were quantified as described in Materials and Methods. (B) Removal of N-terminal palmitoylation does not affect the kinase property. Membrane fractions and soluble fractions of the wild-type (EN-pYPB-GFP-ENV7) and EN-pYPB-C11/14G.ENV7-GFP strains were assayed for kinase activity using bacterially purified GST-Imh1. The upper panel represents the autoradiogram, whereas the lower panel represents the corresponding blot with anti-GFP antibody. (C) Imh1 localization gets disrupted in an *env7* mutant (iIMH-GFP202) compared with the wild type (iIMH-GFP). Fluorescence micrographs of living yeast cells whose genotypes have been indicated above. Punctate structures are represented with white arrows. (D) Immunoblot analysis of the distribution of GFP-Imh1 in the wild type and *env7* mutant. The membrane (Memb) and cytosolic (Cyto) protein fractions isolated from the wild-type (iIMH-GFP) and *env7* mutant (iIMH-GFP202) strains expressing GFP-Imh1 were used for Western blotting. These are two different blots, as indicated by the vertical separation line. (E) Percentage of distribution of Imh1 as membrane associated or cytosolic fractions.

Since Env7 is a predicted serine threonine kinase, we have checked the phosphorylation status of its downstream effector, Imh1 (which has several potential phosphorylation sites at serine threonine residues according to Netphos [http://www.cbs.dtu.dk/services/NetPhos/] and Scansite [http://scansite.mit.edu/]), in the wild type and *env7* mutant. Interestingly, GFP-Imh1p showed reduced intensity of an upshifted band on a low-percentage gel in the *env7* mutant compared with the wild type. This decrease was almost by 15% and was comparable to the decrease seen upon phosphatase treatment (Fig. 6A).

Next we asked whether C11/14G Env7 has ability to interact and phosphorylate Imh1 or whether its mislocalization prevented it from interacting with Imh1 *in vivo*. As an answer to this question, we performed an *in vitro* kinase assay for membrane and cytosolic fractions obtained from strains expressing wild-type and C11/14G versions of Env7. As a substrate, bacterially overexpressed and purified GST-Imh1 was used. As expected, C11/14G versions of Env7 showed kinase activity, and this was comparable to that of the wild type (Fig. 6B). Thus, abrogation of palmitoylation at N-terminal region of Env7 did not affect its kinase activity but only caused the mislocalization of protein (Fig. 2B, right panel). This was also in contrast with *S. cerevisiae* Env7 properties, where N-terminal palmitoylation negatively affected the kinase activity (5).

Furthermore, to elucidate the significance of such interaction, we have checked the localization of Imh1 in the wild type and an *env7* mutant. Interestingly, in the mutant, Imh1 lost its precise Golgi apparatus location: while some percentage of the protein remained associated with the Golgi apparatus, almost an equivalent 60% percentage of protein got distributed throughout the cytoplasm (Fig. 6C and D) as assessed by the built-in software in the microscope (NIS Elements AR). Our experiments thus show that Env7 interacts with Imh1 in a manner that is dependent on the localization of the two proteins but independent of the N-terminal palmitoylation status of Env7.

## DISCUSSION

Env7, a potential serine threonine kinase, is a phosphorylated and palmitoylated protein. This protein is present ubiquitously and has been characterized from few other sources. Ligos et al. (15) reported from a two-hybrid screening that Env7 interacts with an *N*-acetylglucosamine kinase in mammalian systems, although this GlcNAc kinase was not a substrate for Env7. A recent study with the yeast *Saccharomyces cerevisiae* indicated that this protein acts as a negative regulator of membrane fusion (5). However, the molecular details and key players involved in the Env7-mediated functioning are completely unknown. We report the substrate for this ubiquitously present serine-threonine kinase for the first time. Imh1, the only known GRIP domain protein in the Golgi apparatus, has been shown by us to be the direct target for Env7 phosphorylation. This Env7 may not be the sole kinase since we could not find a completely dephosphorylated Imh1p in an *env7* mutant background. Since Golgi apparatus targeting of GRIP domain protein is a multilayer interactive process (16), it had been stressed earlier that a vital finding could be to identify the upstream kinase for Imh1. Taken together, our findings push us a step ahead in understanding the multicomponent Imh1-Arl1 machinery that influences membrane curvature at the *trans*-Golgi network.

### A posttranslationally modified *Ca*Env7 protein forms an integral part of the Golgi trafficking system.

In *Candida albicans*, the major fraction of Env7 localized to the *trans*-Golgi and almost always was detected in the membrane-enriched fraction. A global analysis of protein palmitoylation in yeast reported some new palmitoylated proteins based on the presence or absence of transmembrane domains and the positioning of likely palmitoyl-accepting cysteines. The homologue of Env7 in *S. cerevisiae* was a standalone member in the category of palmitoylated proteins in which the palmitoyl-accepting cysteines are toward the N terminus (10). However, in *C. albicans*, palmitoylation sites were spread across both N and C termini (Fig. 2A; see Fig. S3A and B in the supplemental material); the biological significance of the palmitoylation site toward its C terminus is presently being worked out. Moreover, as mentioned earlier, N-terminal palmitoylation sites do not affect the kinase function of this protein. This kinase function may probably be regulated by the C-terminal cysteine residues that will be taken up in our future studies.

Posttranslational modifications can dramatically influence the function and interactions of proteins and contribute to protein sorting (17). Protein-protein or protein-lipid interactions are affected by phosphorylation that adds a negative charge to the protein, while lipidation increases the hydrophobicity of proteins (18). Under such circumstances, reversible phosphorylation and S-palmitoylation may determine the rate and direction of protein shuttling between intracellular compartments according to physiological needs. Therefore, Env7 in *C. albicans* might be under subtle but tight regulation through interplay between phosphorylation and palmitoylation. The issue of individual palmitoylation sites contributing to the percentage of phosphorylated Env7 also will be taken up by us in future. Since *C. albicans* diverged from the baker’s yeast *S. cerevisiae* several million years ago, the function of several predicted palmitoylation sites in *Ca*Env7 could be quite different from that of *S. cerevisiae*, and to date, genomic studies have revealed significant differences in genomic organization (19, 20) between *Candida albicans* and *Saccharomyces cerevisiae*.

### Imh1 phosphorylation interplay regulates membrane fusion-fission dynamics.

The GRIP domain-containing golgins act as “Velcros” to catch vesicles and keep them near the Golgi apparatus to enhance their eventual fusion (21). Imh1, a GRIP domain-containing protein, is an effector of Arl1and is targeted to the *trans*-Golgi network via interaction with GTP-bound form of Arl1.

**FIG 7:**
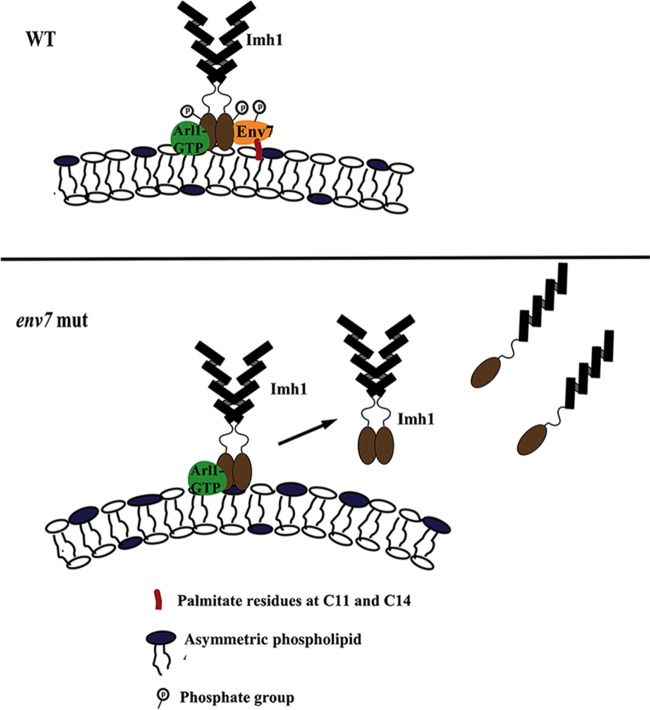
Diagrammatic representation of Env7 functioning at the TGN. Env7 phosphorylation by Env7 is required for maintenance of Imh1 at the membrane. (A) Palmitoylated Env7 tethers to membranes of TGN. Env7p (phosphorylated) phosphorylates Imh1 and keeps it at the site of action (i.e., Golgi membrane). (B) In the *env7* mutant, Imh1 activity is dysregulated and mislocalized from the site of action. Some fraction gets mislocalized to cytoplasm due to conformational changes in the Imh1 tertiary structure, but Arl1 is necessary for initial localization of Imh1 to the Golgi membrane.

A putative working model to maintain membrane asymmetry and curvature at the TGN would be a cooperative networking that involves Arl1, Imh1, and Env7 interaction in an interdependent manner (Fig. 7). Activated Arl1p binds to the C terminus of Imh1p and recruits Imh1 at the TGN (9). Simultaneous phosphorylation of Imh1 by the kinase Env7, the novel partner identified in this study, also seems to be contributing to this site-specific localization and activity of Imh1. We suggest phosphorylation of Imh1 at particular sites by Env7 is important for maintaining Imh1 activity at the TGN; in an *env7* mutant, such a regulatory effect is abrogated and Imh1 probably dysregulated. Such a dysregulated Imh1 becomes unstable and is displaced from its site of action (i.e., the TGN). A displacement from its site of action is accompanied with simultaneous conformational changes that alter anchorage of Imh1 via the GRIP domain at the membrane interface. Thus, a regulated activity of Imh1seems to be important for normal maintenance of membrane curvature, wherein interplay between Env7 and Imh1could be envisaged. Our future studies would focus on the molecular details involved in the phosphorylation-mediated modulation of Imh1 activity and its localization. The mechanistic interplay among Env7, Imh1, and Arl1 at the TGN interface is being worked out in more detail.

### *Ca*Env7 and virulence.

Rapid hypha formation and elongation are crucial for the success of *C. albicans* as a pathogen, so mechanisms that involve secretory pathways are likely to be vital for its fitness and pathogenicity in the host environment. Earlier evidence suggested that virulence is dependent on several properties (22), including the ability to switch between different morphogenetic forms, host epithelial and endothelial cell recognition, and adhesion. These diverse features are, however, united by their dependence on the protein secretory and trafficking apparatus.

The failure of the *Caenv7* mutant to form filaments in solid medium and its tendency to remain in aggregates in YPD liquid medium probably influence its pathogenesis. A regulatory role has also been hypothesized for its mammalian homologue in the control of extracellular matrix cell adhesion. Thus, *Ca*Env7 displays a variety of biological activities defining which could open up new strategies for therapeutic intervention in the human pathogen *C. albicans*. Our studies with the *Candida* homologue of this protein will promote further investigation of this novel protein kinase family, which is involved in an important but poorly defined step in protein trafficking.

## MATERIALS AND METHODS

### Strains and media.

The *C. albicans* strains, described in Table S1 in the supplemental material, were routinely grown on standard yeast extract-peptone-dextrose (YPD) and SD (23) media. Spider medium at 37°C, 2.5 mM GlcNAc in salt base containing 0.45% NaCl, 0.335% yeast nitrogen base (YNB) without amino acids at 37°C (24), or YNB without amino acid and ammonium sulfate with 2% dextrose (SLAD medium) was used for the colony growth assays and also for filamentation induction. For the liquid morphological study, YPD (30°C) was used to pregrow cells to saturation overnight. One percent preculture was added to YPD as bulk culture, and the cells were allowed to grow for 5 to 6 h until they reached the exponential growth phase. Then cells were harvested by centrifugation at 6,000 rpm for 5 min and washed twice with sterile Milli-Q water. Cells were resuspended at an optical density at 600 nm (OD_600_) of 0.5 in various induction media (25). They were induced at 37°C for 4 h in the case of GlcNAc, for 2 h at 37°C in the case of Spider medium and serum, and 2 or 8 h at 30°C in the case of YPD. For the colony growth morphological study, YPD, Spider (26), SLAD, and minimal SD plates were used for the colony growth assays. Sensitivity to Congo red and calcofluor white was tested by spotting dilutions of cells onto YPD plates prepared with the indicated concentration of the corresponding chemical. All strains and plasmids used are listed in Table S1 in the supplemental material. For colocalization studies with *trans*-Golgi (Sec7) and *cis*-Golgi (Vrg4) markers, strains BES and BEV were prepared. To prepare BES, BWP17 was sequentially transformed with pYPB-ENV7.GFP and CIP.mCherry.SEC7 plasmids. Similarly strain BEV was prepared by transforming BWP17 sequentially with pYPB-ENV7.GFP and CIP-mCherry.VRG4 plasmids.

10.1128/mSphere.00080-16.4 TABLE S1  Strains and plasmids used in this study. Download TABLE S1, DOC file, 0.1 MB. Copyright © 2016 Rao et al. 2016 Rao et al. This content is distributed under the terms of the Creative Commons Attribution 4.0 International license.

10.1128/mSphere.00080-16.5 TABLE S2  List of primers used in this study. Download TABLE S2, DOCX file, 0.01 MB. Copyright © 2016 Rao et al. 2016 Rao et al. This content is distributed under the terms of the Creative Commons Attribution 4.0 International license.

### Preparation of *CaENV7* mutant.

The *CaENV7* (orf19.7164) deletion was constructed in *C. albicans* strain SN152 using a method described previously (27, 28). Integration of the deletion cassettes at the appropriate sites was verified by PCR using combinations of primers that flanked the integration as well as primers that annealed within the cassettes that had been introduced (CHECK-MUT1, CHECK-LEU1, and CHECK-HIS1). Complemented strains were constructed by introducing a plasmid carrying one wild-type copy of *CaENV7* into the RP10 locus of genome.

The *CaENV7* gene was initially disrupted by the URA Blaster method. Concerns regarding the positional effect of *URA* on virulence and morphogenesis prevented us from using this mutant for the above mentioned studies, but the double mutant generated by this method was routinely used to transform overexpression plasmids. The plasmid pUC19-CUB carrying a *Candida* URA Blaster cassette, consisting of the *C. albicans* URA3 gene flanked by *hisG* sequences, was used from laboratory glycerol stock. Plasmid pE7, which had the *CaENV7* open reading frame (ORF) along with 1-kb upstream and downstream fragments, was sequentially digested with the XhoI and EcoRV enzymes and finally ligated to pUC19-CUB digested with SalI and SmaI, which released the URA Blaster cassette. *CaENV7* was disrupted in strain CAI-4 by sequential gene replacement and recycling of the *URA3* marker by selection on SD plus 5-fluoroorotic acid (1 mg/ml) and uridine (50 µg/ml). A reintegrant strain was also constructed in which a wild-type copy of *CaENV7* was transformed into the null mutant.

### TAP tagging.

To tandemly tag *Ca*Env7p with the HF (6×His and 6×Flag) epitope in the genomic locus, a DNA fragment containing the 3′ region of *ENV7*, the 6×HF tag sequence, the ACT1 terminator, the *URA3* marker, and the downstream region of *ENV7* was amplified, using p6HF-ACT1 (29) as the template and ENV-HF-F and ENV-UR-R1 as the primers and introduced into BWP17 to generate iENV7-HF.

### TAP.

*Ca*Env7-TAP purification was performed as described previously (30, 31). The strains BWP17 and iENV7-HF were grown at 30°C to an OD_600_ of 2 and harvested by centrifugation. Cell pellets (1 liter of culture) were washed once with ice-cold water and twice with yeast extract buffer (YEB) without protease inhibitors and snap-frozen in liquid nitrogen. To make 300 ml of buffer, 36.6 ml of 2 M KCl was mixed with 3 ml of 0.5 M EDTA, 3 ml of 0.5 M EGTA-KOH (pH 7.9), and 30 ml of 1 M HEPES-KOH (pH 7.9) in 229 ml of double-distilled water. The solution was stored at room temperature. Prior to use, 750 µl of 1 M dithiothreitol (DTT) was added. The frozen pellet was ground to fine power in a mortar and pestle and collected in 50-ml centrifuge tubes. This fine powder was completely dissolved in an equal volume of YEB containing 0.1% Triton X-100, a protease inhibitor cocktail (Complete EDTA-free tablet [Roche]), PhosphoSTOP, and 1 mM phenylmethylsulfonyl fluoride (PMSF [Roche PMSF Plus]) on a rocker shaker. Then, the cell lysate was subjected to ultracentrifugation at 45,000 rpm for 1 h at 4°C. The clarified cell lysates were first subjected to anti-FLAG M2 affinity agarose (A2220 [Sigma’) (15 to 20 mg of protein with 60 to 80 µl of beads) for 4 h followed by buffer exchange to phosphate buffer (NaH_2_PO_4_ [pH 8.0], 150 mM NaCl, 0.25% Nonidet P-40 containing protease inhibitor cocktail with Complete EDTA-free tablet, and PMSF [Roche, Germany]). The first-round elute was then applied to Ni-nitrilotriacetic acid (NTA) agarose (30210 [Qiagen]), incubated for 1 h, followed by second round of elution with 300 mM imidazole. After a second round of purification, the residual agarose beads were removed by passage of the eluate through Ultrafree-MC centrifugal filter units (Millipore, MA). The eluant was boiled with 1× SDS gel loading buffer and loaded on a 10% SDS-PAGE gel. The protein bands were detected by staining with silver stain (Bio-Rad).

### Coimmunoprecipitation experiments.

Cells expressing N-terminally GFP-tagged *IMH1* in the background of 13×Myc-tagged *ENV7* and strain BWP17 were grown in YP galactose medium, to which 0.6 M KCl was added, and induced for 30 min once the cells reached the exponential phase. Total extracts were prepared from 500 ml of culture that was pelleted, washed with HEPES buffer without inhibitors, and resuspended in immunoprecipitation (IP) buffer (HEPES buffer) containing Roche Complete EDTA-free protease inhibitor and PhosphoSTOP (Roche). For the IP with anti-GFP antibody (ab290 [Abcam]), the lysis was performed in the presence of glass beads by vortexing the cells for 8 min with a rest of 1 min in between each cycle at maximum speed at 4°C. The cell debris was eliminated by centrifugation (20 min at 4°C and 13,000 rpm). For immunoprecipitations, equal bead volumes of protein A Sepharose (P9424 [Sigma]) and protein G Sepharose (P3296 [Sigma]) were mixed, washed three times with HEPES buffer containing 0.25% NP-40, and aliquoted. Cell lysates were precleared with 20 µl of bead mixture for 1 h at 4°C. Precleared lysates containing 5 mg of protein were then incubated with 3 µl of anti-GFP antibody for 2 h at4°C, which was finally added to 30 µl of bead mixture and incubated for 4 h at 4°C. Then beads were washed three times with HEPES buffer. Immunoprecipitated proteins were released from the beads by boiling them for 5 min. Eluted proteins were separated by SDS-PAGE and analyzed by Western blotting with anti-c-Myc antibody (13585700 [Roche]) or anti-GFP antibody separately. As input, 5% crude lysates was included in the gel.

### Total membrane protein isolation.

Yeast total membrane proteins were isolated as described by Lenoir et al. (32) with slight modifications. Yeast strains were induced in YPD with 0.6 M KCl.

### Membrane fractionation.

Subcellular fractionation was carried out as described by Cabrera et al. (30). Cell pellets were washed and were treated with 10 mM DTT followed by an incubation with lyticase for 20 min at 30°C. Spheroplasts were resuspended in 1 ml of lysis buffer containing 2 µg/ml DEAE-dextran and incubated on ice for 5 min, followed by a 2-min incubation at 30°C. The total extract was centrifuged twice at 300 × *g* for 3 min at 4°C to removed unlysed cells, and the supernatant (S4) was then centrifuged at 13,000 × *g* for 15 min to isolate the pellet (P13) used for checking the Env7-upshifted phosphorylated band estimation in an *in vitro* kinase assay.

### Palmitoylation assay.

The palmitoylation assay was carried out according to a method described previously (33) with modifications. The beads were washed with ABE buffer containing 0.1% Triton X-100 and incubated with 50 mM *N*-ethylmaleimide (NEM) in ABE buffer for 1.5 h. They were washed and incubated with either 1 M Tris (pH 7.5) (control), or 1 M hydroxylamine (pH 7.5) for 2.5 h at room temperature and analyzed by Western blotting. In another set of experiments, the P13 fraction of WT Env7-GFP was treated with phosphatase (100 U), phosphatase inhibitors (1 µM sodium orthovanadate, 1 µM β-glycerophosphate, and 50 µM sodium azide), or 1 M hydroxylamine and analyzed by Western blotting. For phosphorylation upshifts, bands of each protein species were densitometrically quantified as described below, and the extent of phosphorylation (upshifted species) was expressed as a percentage of the total (upshifted + non-upshifted) for each protein.

### Densitometric scanning.

Bands from lower-exposure blots were densitometrically scanned using Vision Works LS Image acquisition and analysis software version 6.8(Life Science Software from UVP) and corrected by subtracting the corresponding background area of a blot.

### Site-directed mutagenesis.

Site-directed mutagenesis was carried out to change the predicted cysteine residues for palmitoylation to glycine residues using QuikChange site-directed mutagenesis kits (Stratagene catalogue no. 200518) using primers ENV-SDM-F and ENV-SDM-R, and the product was transformed into XL-1 Blue supercompetent cells. The positive colony was confirmed by sequencing and finally transformed in the *Caenv7* mutant.

### Western blot analysis.

For Western blot analysis, 25 to 60 µg of proteins from gels was electrotransferred to Hybond C Extra membrane (Amersham Biosciences). The blots were processed as described by Rao et al. (25).

### Yeast two-hybrid analysis.

Yeast two-hybrid analysis was done as described elsewhere (34) and following manufacturer’s instructions. Yeast transformations were done in four sets with 2 µg of plasmid DNA of pGAD424-Imh1, pGBT9-Env7, or pCL1 alone and pGAD424-Imh1 and pGBT9-Env7 together. One-third of the transformation mixes of pGAD424-Imh1 and pCL1 were plated on SD dropout −Leu plates, that of pGBT9-Env7 on SD dropout −Trp plates, and that of pGAD424-Imh1 and pGBT9-Env7 together on SD dropout −Trp and −Leu plates, respectively, for the selection of transformants.

### Kinase assay.

For kinase assays of Env7-GFP, the whole membranous fractions (P13) were used (5, 30). The membranes were solubilized by resuspending them in lysis buffer containing 1% Triton X-100 and 500 mM NaCl, incubated on ice for 30 min, and centrifuged at 13,000 × *g* for 30 min to obtain solubilized Env7-GFP, which was then immunoprecipitated overnight at 4°C. Kinase activity was assayed in a total reaction volume of 20 µl that contained 10 µl of immunoprecipitated beads, 200 µM of unlabeled ATP, 1 mg/ml bovine serum albumin (BSA), and 10 µCi [γ-^32^P]ATP with or without the addition of 2.5 µg purified GST-Imh1. The kinase reaction was carried out by incubating the reaction mixture at 30°C for 30 min and stopped by the addition of 5 µl of 4× SDS-PAGE sample buffer. The solubilized proteins were heated at 100°C for 5 min and resolved by 10% SDS-PAGE.

### Phosphatase treatment.

The P13 membrane fractions of WT and ENV7-GFP per 40 µg of crude protein extracts from iIMH-GFP and iIMH-GFP202 strains were treated with 100 U of lambda protein phosphatase (NEB catalogue no. P0753S) following the manufacturer’s instructions and analyzed by Western blotting.

### Microscopy.

Microscopic images of yeast cells were captured using a Nikon 80i inverted microscope equipped with a Nikon digital DXM1200C camera and stereo microscope. Images were processed using Adobe Photoshop version 5.5 (Adobe Systems, Corp., San Jose, CA). Fluorescence images were captured by using a Nikon A_1_R confocal laser scanning microscope.

### Electron microscopy.

The iENV7-Myc strain was grown to the exponential phase before being processed for electron microscopy by following the procedure described elsewhere (35) with minor modifications as briefly described here. Washed cells (in 0.1 M phosphate buffer) were fixed for 2 h (4% paraformaldehyde plus 0.5% glutaraldehyde in 0.1 M phosphate buffer) following aldehyde quenching (100 mM ammonium chloride in phosphate buffer). Then cells were resuspended in 2.5% molten agar to prepare 0.5-mm thin slices before being subjected to dehydration through ethanol series in water/LR White resin (50 to 100%). Then samples were infiltrated at room temperature with LR White resin (Sigma catalogue no. L9774). Resin was kept at 55°C in vacuum oven for 72 h for polymerization. Cut sections of uniform thickness (90 nm) were collected on nickel grids (200 mesh [Electron Microscopy Sciences]) and incubated with mouse monoclonal anti-c-Myc antibody (Roche) (1:3 dilution) for 16 h at 4°C, followed by washing and incubation with colloidal gold-conjugated secondary antibody (1:100 dilution) for 1 h. The grids were stained with uranyl acetate (saturated solution of uranyl acetate in 50% ethanol) for 5 min before being visualized under electron microscope.

### Murine model of hematogenously disseminated candidiasis.

Eight- to 10-week-old female BALB/c mice (*Mus musculus*) (18 to 20 g) were used for the experiments. Mice were challenged intravenously on day 0 with 10^6^ CFU in 200 µl phosphate-buffered saline (PBS). In order to assess the fungal burden in kidney tissue, the dose described above was injected (5 mice per strain per experiment), and mice were sacrificed at various time points (24, and 72 h) postinfection. Kidneys from individual mice were removed aseptically, weighed, and homogenized in 1-ml PBS buffer by using a homogenizer. CFU counts were determined by plating serial dilutions on YPD agar medium. The colonies were counted after 24 h at 37°C, and the results were expressed as log CFU per gram of tissue.

The animals were handled in strict accordance with the principles outlined by the Institutional Animal Ethics Committee (Jawaharlal Nehru University, New Delhi, India).
