# Supplementary figures and images for: Env7p Associates with the Golgin Protein Imh1 at the trans-Golgi Network in Candida albicans
Source: mSphere. 2016 Aug 3;1(4):e00080-16. doi: 10.1128/mSphere.00080-16 (PMC4973633; doi:10.1128/mSphere.00080-16)

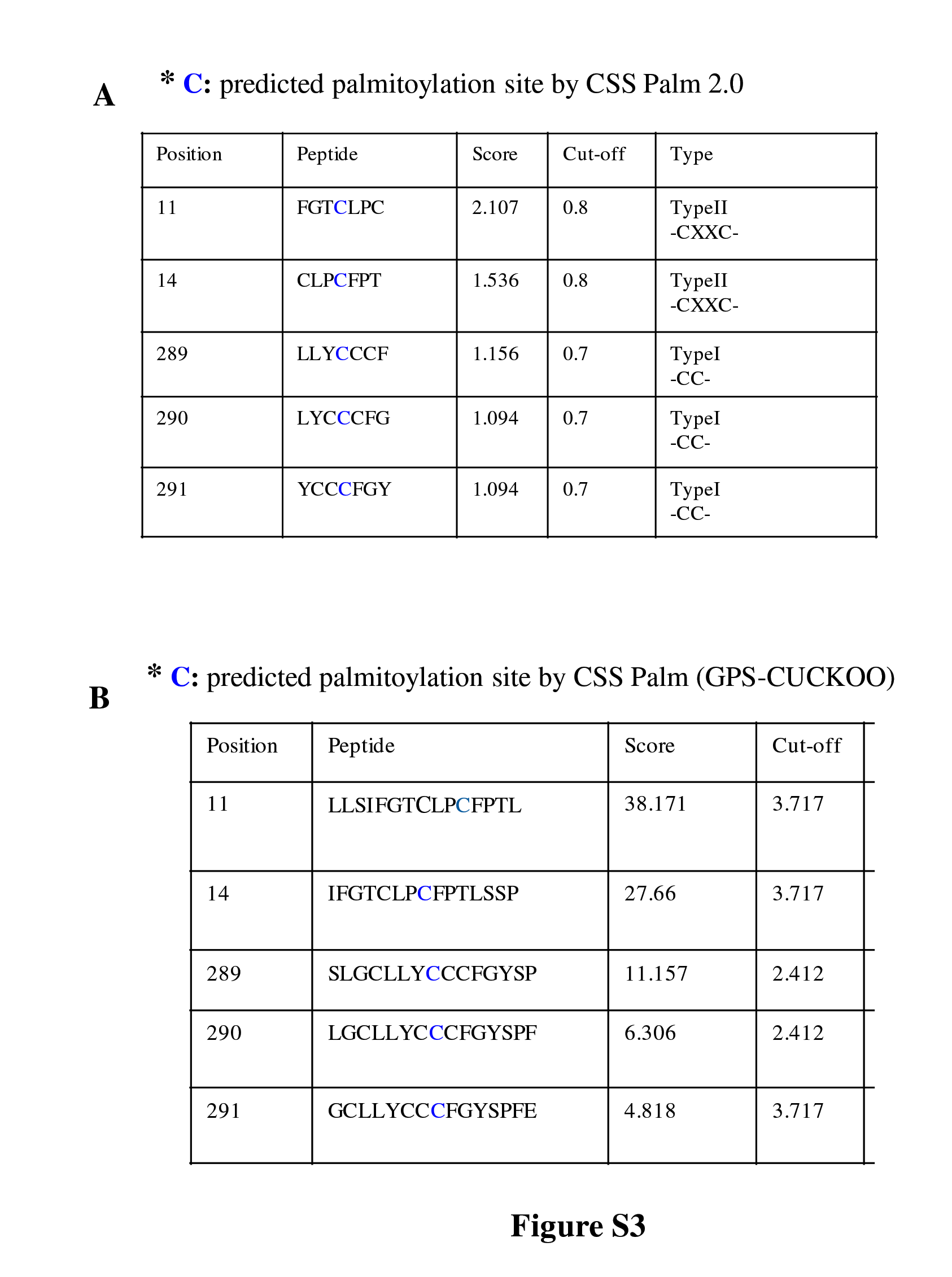

Supplement: FIGURE S3 [file sph004162119sf5.tif]
