# Supplementary material for: Env7p Associates with the Golgin Protein Imh1 at the trans-Golgi Network in Candida albicans
Source: mSphere. 2016 Aug 3;1(4):e00080-16. doi: 10.1128/mSphere.00080-16 (PMC4973633; doi:10.1128/mSphere.00080-16)
Supplement: TABLE S1 [file sph004162119st1.doc]

**Table S1. Strains and plasmids used in this study**

| **Strains** | **Relevant genotype and description** | **Source or reference** |
| --- | --- | --- |
| ***Candida albicans***  SN152  EN101  EN102  EN103  SN-HLA  SC5314    CAF2-1  CAI4  EN201  EN202  EN203  iENV7-GFP  iENV7-Myc  EN-pYPB-ENV7-GFP  EN-pYPB-C11/14G.ENV7-GFP  EN-pYPB- GFP-ENV7  CAI-pYPB  IpGAL-ENV7  BWP17  BES  BEV  iENV7-HF  BE7  BEI71  BEI71-C11/14G  iIMH-GFP  iIMH-GFP202  ***E.Coli* Srains**  DH5α  XL-1Blue  **Plasmids**  pUC19  pCUB6  pUC19-CUB  pE7  pE7-hUh  pCa*ENV7*  p6HF-Act1  pMYC.HIS1  pYFP.URA3  pURA3.PGAL1.GFP  pYBP.ADH1pt  pYBP.ADH1.ENV7.GFPpt  pYBP.ADH1C11/14G.ENV7.YFPpt  pYBP.ADH1.GFP.ENV7 pt  CIP-ADH1.mCherry.SEC7  CIP-ADH1.mCherry.VRG4  pGBT9  pGBT.ENV  pGBT.C11/14G ENV  pGAD424  pGAD.IMH | *arg4Δ/arg4Δleu2Δ/leu2his1Δ/his1Δ URA3/ura3Δ::imm434*  *IRO1/iro1 Δ::imm434*  *SN*152, *ENV7/env7*::*HIS1*  *EN101,env7*:*:LEU2 /env7::HIS1*  EN102, *env7*:*:LEU2 /env7::HIS1/ENV7-ARG4*  *arg4Δ/ARG4Δleu2Δ/LEU2his1Δ/HIS1URA3/ura3Δ::imm434*  *IRO1/iro1 Δ::imm434*  *URA3*/*URA3*  *Δura3*::*imm434/URA3*  Δ*ura3*::*imm434*/Δ*ura3*::*imm434*  CAI4, *env7*Δ::*hisG/env7*Δ::*hisG-URA3-hisG*  *EN201, env7*Δ::*hisG/env7*Δ::*hisG*  *EN202, env7*Δ::*hisG/ENV7-URA3*  *CAI4, CaENV7-GFP1-ADH1T-URA3/CaENV7*  *CAI4, CaENV7-13xMyc- ADH1T-URA3/CaENV7*  *EN202* transformed with pYPB-ENV7.GFP plasmid  *EN202* transformed with pYPB-C11/14G.ENV7.GFP plasmid  *EN202* transformed with pYPB- GFP.ENV7 plasmid  *CAI-4* transformed with pYPB-ADH1-pt plasmid  *CAI4, pGAL1.ENV7::URA3*  *ura3*_::*limm434*/*ura3*_::*limm434 his1*::*hisG/his1*::*hisG arg4*::*hisG/arg4*::*hisG*  *BWP17* transformed with pYPB-ENV7.GFP and CIP.mCherry.SEC7 plasmids  *BWP17* transformed with pYPB-ENV7.GFP and CIP-mCherry.Vrg4 plasmids  *BWP17 plus CaENV7-6HF- ADH1T-URA3/CaENV7*  *BWP17 plus ENV7-13XMYC*::*HIS1*  *BWP17 plus ENV7-13XMYC*::*HIS1, pGAL1.GFP-IMH1::URA3*  *BWP17 plus* C11/14G *ENV7-13XMYC*::*HIS, pGAL1.GFP-IMH1::URA3*  *CAI4, pGAL1.GFP-IMH1::URA3*  *EN202 plus pGAL1.GFP-IMH1::URA3*  F’*/endA1 hsdR17*(r_K_^-^m_K_^+^) *gln*V44 *thi-1 rec*A1 *gyrA (Nal ^r^) relA Δ(laclZYA-argF)*U169 *deoR(φ80dlacΔ(lacZ)M15)*  *recA1 endA1 gyrA96 thi-1 hsdR17 supE44 relA1 lac* [F´ *proAB lacI*q*Z*Δ*M15* Tn*10* (Tetr)].  Cloning vector  *Cloned C.albicans* URA blaster cassette  “*hisG-URA3-hisG*” cassette cloned in pUC19  *ENV7* cloned in pUC19  “*hisG-URA3-hisG*” cassette cloned in pE7  ENV7 ORF plus 1Kb Upstream and 300bp down stream  Vector containing 6XHIS and FLAG sequence with URA3  Vector containing 13XMYC sequence with HIS1 marker  Vector containing YFP sequence with URA3 marker  Vector containing N-terminal GFP seq under GAL1 promoter  C.albicans expression vector contains ADH1 promoter and terminator sequences  ENV7.GFP cloned in pYBP.ADH1pt  C11/14G ENV7.YFP cloned in pYBP.ADH1pt  GFP.ENV7 cloned in pYBP.ADH1pt  SEC7 cloned in CIP-ADH1.mCherry plasmid  VRG4 cloned in CIP-ADH1.mCherry plasmid  Y2H vector containing GAL4 DNA binding domain  ENV7 ORF sequence cloned in pGBT9  C11/14G ENV7 ORF sequence in pGBT9  Y2H vector containing GAL4 Activation domain  IMH1 ORF sequence cloned in pGBT9 | Alexander D. Johnson  This study  This study  This study  This study  W.A. Fonzi  W.A. Fonzi  W.A. Fonzi  This study  This study  This study  This study  This study  This study  This study  This study  This study  This study  Jim Dover  This study  This study  This study  This study  This study  This study  This study  This study  Invitrogen Life Technology  Stratagene  NEB  W. A. Fonzi  Asis Datta  This study  This study  This study  Masakazu Niimi  This study  Judith Berman  Judith Berman  AJP Brown  This study  This study  This study  Under communication  Under communication  ClonTech  This study  This study  ClonTech  This study |
