# Supplementary material for: Env7p Associates with the Golgin Protein Imh1 at the trans-Golgi Network in Candida albicans
Source: mSphere. 2016 Aug 3;1(4):e00080-16. doi: 10.1128/mSphere.00080-16 (PMC4973633; doi:10.1128/mSphere.00080-16)
Supplement: TABLE S2 [file sph004162119st2.docx]

**Table S2. List of primers used in this study.**

| S.NO | Name | Sequence |
| --- | --- | --- |
| 1  2  3  4  5  6  7  8  9  10  11  12  13  14  15  16 | ENV-GF-F  ENV-Myc-F  ENV-HF-F  ENV-UR-R  ENV-AD-GF1  ENV-AD-R  ENV-SDM-F  ENV-SDM-R  CHECK-SEQ-1  ENV7 -DEL –F  ENV7-DEL-R  CHECK-MUT-1  CHECK-LEU-1  CHECK-HIS-1  IM-EX-F2  IM-EX-F2 | 5’GTTTACAATTAAACCCAGACAGTAGACCTGATATAGACGAATTGTTAAATGACGTCTTGGAATTAACGAGACAGTTAGGAACGGGTGGTGGTGGTTCTAAAGGTGAAGAATTATT 3’  5’GTTTACAATTAAACCCAGACAGTAGACCTGATATAGACGAATTGTTAAATGACGTCTTGGAATTAACGAGACAGTTAGGAACGGGT GGTGGTCGGATCCCCGGGTTAATTAA 3’  5’GTTTACAATTAAACCCAGACAGTAGACCTGATATAGACGAATTGTTAAATGACGTCTTGGAATTAACGAGACAGTTAGGAACGGGT CTCGAGGGTGCATGCCAC 3’  5’TTGTATTTTTACCGCAAATCCGCACAATACATTCTATACACCTATACTGCCAGAGAAATTTTAAAGCAACTTAAATGCATGTGTATTCTAGAAGGACCACCTTTGATTG3’  5’ACCCGGATCCAGACATGGATGTATTAC3’  5’GTGTGGATCCATTTACGTTCCTAACTG3’  5’ACTATCGATTTTGGAACT**G**GCTTGCCT**G**GCTTCCCCACTTT3’  5’AAAGTGGGGAAGC**C**AGGCAAGC**C**AGTTCCAAAAATCGATAGT3’  5’GATTATCAGATTACTACGCC3’  5’GACTACAACAACACAAAGTCAACTATAACATTTCATTTTTCAATCAGTCAAACTATCACTACTATATCACCTACCCAGACAGCTCGGATCCACTAGTAACG 3’  5’TTGTATTTTTACCGCAAATCCGCACAATACATTCTATACACCTATACTGCCAGAGAAATTTTAAAGCAACTTAAATGCATGTGTATGCCAGTGTGATGGATATCTGC 3’  5’TACACCTTCAAACAAGCAATT 3’  5’TACATGCAGAACCAGAACTC 3’  5’TAGTTATTTCTTCGGTTCTTTC 3’  5’AGTATCTATCAATTTAAATTGAAAAAATAAAGAACTACCTTGAAACACAATTTCACCTTCTAGAAGGACCACCTTTGATTG 3’  5’GATCAGACAAGTTATTCAACTCATTGATCACATCTTCTGAGAAATTCTTAAGTTTCGAAAATTTGTACAATTCATCCATAC 3’ |
